# An improved version of systematic sampling design for use with linear trend data

**DOI:** 10.1016/j.heliyon.2023.e17121

**Published:** 2023-06-08

**Authors:** Muhammad Azeem, Sundus Hussain, Musarrat Ijaz, Najma Salahuddin, Abdul Salam

**Affiliations:** a Department of Statistics, University of Malakand, Chakdara, Khyber Pakhtunkhwa, Pakistan; b Department of Statistics, Shaheed Benazir Bhutto Women University, Peshawar, Pakistan; c Department of Statistics, Rawalpindi Women University, Rawalpindi, Pakistan

**Keywords:** Efficiency, Sample surveys, Diagonal systematic sampling, Milk yield data, Linear trend, **Mathematics Subject Classification 2020**, 62D05, 65C05

## Abstract

In survey sampling, systematic sampling design has attracted survey researchers in recent years due to its simplicity of use. We introduce a modified variant of systematic sampling scheme which improves the efficiency of a recently developed diagonal systematic sampling method. The suggested modification is also found to be more efficient than the other popular sampling designs in circumstances where the units of the population under consideration exhibit an increasing or decreasing perfect or near-perfect linear trend. Moreover, it is observed that the conditions for efficiency are mathematically strong and practically always hold, hence making the suggested sampling design preferable over the available sampling designs.

## 1 Introduction

In recent decades, survey researchers have taken a keen interest in systematic random sampling as it is an easy but useful procedure of selection of a random sample from a given population of interest. Researchers find systematic sampling even simpler than the default simple random sampling due to the fact that it selects only the first unit (or the first few units) randomly from the population of interest. The remaining units for the sample are obtained by using a pre-defined rule. First introduced by Madow and Madow [1], systematic random sampling and its various forms are used by the researchers under different real-life circumstances. Madow and Madow [1] introduced the novel idea of a pre-defined pattern for selection of units and termed it systematic sampling. Madow and Madow [1] method was only manageable in circumstances where the size of the finite population can be regarded as a constant multiple of the sample size, which limits its usability. To alleviate this problem, Lahiri [2] presented the circular type of systematic sampling design. Later on, Chang and Huang [3] suggested a modification on the systematic random sampling scheme which they called remainder systematic sampling design which can be applied in situations in which the size of a finite population cannot be expressed as a multiple of the size of the sample. Subramani [4] introduced what is known as diagonal-systematic sampling. As its name suggests, the units are obtained diagonally in diagonal systematic sampling. Sampath and Varalakshmi [5] as well as Subramani [6] introduced modified forms of the diagonal systematic sampling design. For those cases with the sample size being an odd integer, Subramani [7] introduced an efficient form of the linear systematic sampling design which was found to be more efficient than the previous versions of systematic sampling. Likewise, another efficient form of the linear systematic sampling design was introduced in the research study of Subramani and Gupta [8]. The Subramani and Gupta [8] technique was beneficial as it didn't require a mathematical relationship between the population and sample size. More recently, Azeem and Khan [9] studied the estimation of mean under a new modification on the diagonal systematic sampling scheme. In addition, several other survey researchers also studied different aspects of the variants of systematic random sampling based on real-life situations, including the research studies of [[10], [11], [12], [13], [14], [15], [16], [17], [18], [19], [20], [21]].

Azeem et al. [22] suggested a new modified variant of the diagonal systematic random sampling and proved the improvement in terms of efficiency over both linear and diagonal systematic sampling, in addition to some of the other available sampling schemes. Motivated by the study of Azeem et al. [22], we introduce a modified form of the Azeem et al. [22] sampling method. We observe that our proposed version is more efficient than Azeem et al. [22] sampling scheme. We also show the improvement over some other popular sampling schemes for those real-life situations where a linear trend exists among the units of the population. The sampling variance of the mean on the basis of the new suggested sampling design is derived. The improvement in efficiency is observed for a real data set as well as for situations with a perfect linear trend..

**Table 1:** Population units organized in different sets.

Set-I					Set-II				
S.No.	1	2	…	k	S.No.	1	2	…	k
1	$y_{1}$	$y_{2}$	…	$y_{k}$	*k*+1	$y_{kk+1}$	$y_{kk+2}$	…	$y_{kk+k=\left(k+1\right)k}$
2	$y_{k+1}$	$y_{k+2}$	…	$y_{2k}$	*k*+2	$y_{\left(k+1\right)k+1}$	$y_{\left(k+1\right)k+2}$	…	$y_{\left(k+2\right)k}$
3	$y_{2k+1}$	$y_{2k+2}$	…	$y_{3k}$	*k*+3	$y_{\left(k+2\right)k+1}$	$y_{\left(k+2\right)k+2}$	…	$y_{\left(k+3\right)k}$
k	$y_{\left(k-1\right)k+1}$	$y_{\left(k-1\right)k+2}$	…	$y_{kk}$	*n*-2	$y_{\left(n-3\right)k+1}$	$y_{\left(n-3\right)k+2}$	…	$y_{\left(n-2\right)k}$
**Set-III**									
S.No.	1	2	…	*k*					
*n-*1	$y_{\left(n-2\right)k+1}$	$y_{\left(n-2\right)k+2}$	…	$y_{\left(n-1\right)k}$					
*n*	$y_{\left(n-1\right)k+1}$	$y_{\left(n-1\right)k+2}$	…	$y_{nk}$					

## 2 Suggested sampling design

Suppose the finite population under consideration consists of *N* units and let a random sample of size *n* is needed to be obtained such that N=kn=kk+(n−k−1)k+k. The new proposed method selects the sample from the finite population in the following steps:

1) Partition the entire population into three non-overlapping and exhaustive sets of units: Set-I, Set-II and Set-III, so that Set-I gets the first $k\times k=k^{2}$ units $y_{i}$ (*i* = 1, 2,…, $k^{2}$) to form a k×k square matrix, Set-II gets the next (n−k−2)k units $y_{i}$ (i=kk+1,kk+2,kk+3,...,(n−2)k), whereas Set-III gets the last 2*k* units $y_{i}$ (i=(n−2)k+1,(n−2)k+2,(n−2)k+3,...,nk).
2) Arrange the units in Set-I in a k×k matrix of units. Inside Set-II, place the (n−k−2)k units in the form of a matrix having order (n−k−2)×k, and in Set-III, organize the last 2*k* units in a matrix of order 2×k, as given in Table 1.
3) Select three random numbers $r_{1}$, $r_{2}$ and $r_{3}$ where $1\leq r_{1}\leq k$, $1\leq r_{2}\leq k$ and $1\leq r_{3}\leq k$. In Set-I, the units for the sample are chosen in such a manner that the chosen k units belong to the diagonal of the resulting matrix of units. In Set-II, units for the sample are chosen in a manner so that the chosen n−k−2 units belong to the $r_{2}$ th column of the matrix. Likewise, in Set-III, two units are randomly selected from the total 2*k* units in the set in such a manner that the selected units belong to the same column. Finally, the units selected from all three sets are merged into a single group, hence yielding the required sample of size *n.*

It is to be noted that the suggested method differs from the one suggested by Azeem et al. [22] method in the sense that it partitions the finite population into three non-overlapping groups as opposed to the two groups in the Azeem et al. [22] method. That is, the last two rows of Set-II in the Azeem et al. [22] have been moved into a separate group – named Set-III. Like the Azeem et al. [22] method, the allocation of units to the three sets is done in a mutually exclusive and collectively exhaustive way in the proposed method too. In Section 4, it is observed that this approach of unit-allotment results in a more improved sampling design.

The proposed sampling scheme divides the population into three non-overlapping groups, which leads to a more efficient sampling design compared to the available sampling schemes which are based on dividing the population into two groups. It is to be noted that the population may be divided into more than three groups for further improvement in efficiency over the suggested method, however, it may result in a very limited number of choices between the values of *N* and *n*, which may not be practically applicable. Thus, the division of population into three groups is a good balance between efficiency and the practical usefulness of the sampling scheme.

One can easily observe that the total number of possible samples that can be selected in the new sampling design is $k\times k\times k=k^{3}$, each having size *n*. The first order probability of inclusion based on the suggested sampling design is given by:

(1) $$\pi_{i}=\frac{1}{k}$$

where the subscript ‘*i*’ denotes the *i*th unit of the population. Also,

(2) $$\pi_{ij}=\left\{ \begin{array}{c} \frac{1}{k},if\;ith\;and\;jth\;units\;belong\;to\;the\\ \text{same}\;diagonal\;\text{in}\;Set-I,\\ \frac{1}{k},if\;ith\;and\;jth\;units\;belong\;to\;the\\ same\;column\;of\;Set-\text{II}\;\text{or}\;\text{Set}-\text{III},\\ \frac{1}{k^{2}},if\;ith\;and\;jth\;units\;are\;from\\ \text{two}\;\text{different}\;\text{sets},\\ 0,otherwise. \end{array}\right.$$

Generally, the units selected under the proposed sampling scheme are:

$$S_{r_{1}r_{2}r_{3}}=\left\{ \begin{array}{l} y_{r_{1}},y_{\left(k+1\right)+r_{1}},...,y_{\left(k-1\right)\left(k+1\right)+r_{1}},y_{kk+r_{2}},y_{\left(k+1\right)k+r_{2}},...,y_{\left(n-3\right)k+r_{2}},y_{r_{3}},y_{k+r_{3}}.\;if\;r_{1}=1\\ \begin{array}{l} y_{r_{1}},y_{\left(k+1\right)+r_{1}},...y_{t\left(k+1\right)+r_{1}},y_{\left(t+1\right)k+1},y_{\left(t+2\right)k+2},...,y_{\left(k-1\right)k+k-t-1},\\ y_{kk+r_{2}},y_{\left(k+1\right)k+r_{2}},...,y_{\left(n-2\right)k+r_{2}},y_{r_{3}},y_{k+r_{3}}. \end{array}\}\;if\;r_{1}>1 \end{array}\right.$$

where $r_{2}=1,2,...,k$, $r_{3}=1,2,...,k$.

The mean on the basis of the new suggested sampling design is given by:

(3) $${\bar{y}}_{msy}=w_{1}{\bar{y}}_{1}+w_{2}{\bar{y}}_{2}+w_{3}{\bar{y}}_{3}\text{,}$$

where the mean of the sample from Set-I is given in equation (4) as:

(4) $${\bar{y}}_{1}=\left\{ \begin{array}{l} \frac{1}{k}\sum_{l=0}^{k-1}y_{l\left(k+1\right)+r_{1}},\text{if}\;r_{1}=1,\\ \frac{1}{k}\left(\sum_{i=0}^{t}y_{i\left(k+1\right)+r_{1}}+\sum_{i=1}^{k-t-1}y_{\left(t+i\right)k+i}\right),\text{if}\;r_{1}>1. \end{array}\right.$$

where *t* = *k - r*_*1*_. The mean of the sample from Set-I and Set-II are given in equations (5), (6) as:

(5) $${\bar{y}}_{2}=\frac{1}{n-k-2}\sum_{l=k}^{n-3}y_{lk+r_{2}},w_{1}=\frac{k}{n},w_{2}=\frac{n-k-2}{n},w_{3}=\frac{2}{n},w_{1}+w_{2}+w_{3}=1\text{,}$$

and,

(6) $${\bar{y}}_{3}=\frac{1}{2}\left(y_{\left(n-2\right)k+r_{3}}+y_{\left(n-1\right)k+r_{3}}\right)\text{.}$$

Theorem: The mean of the sample may be expressed in the mathematical form given by Horvitz and Thompson [23] and is unbiased estimator of the population mean with variance given as:

$$Var\left({\bar{y}}_{msy}\right)=\frac{1}{N^{2}}[k^{4}\left\{\frac{1}{k}\sum_{i=1}^{k}{\left({\bar{y}}_{1i}-{\bar{Y}}_{1}\right)}^{2}\right\}+{\left(n-k-2\right)}^{2}k^{2}\left\{\frac{1}{k}\sum_{i=1}^{k}{\left({\bar{y}}_{2i}-{\bar{Y}}_{2}\right)}^{2}\right\}+4k^{2}{\left({\bar{y}}_{3i}-{\bar{Y}}_{3}\right)}^{2}]\text{,}$$

where ${\bar{y}}_{1}$, ${\bar{y}}_{2}$ and ${\bar{y}}_{3}$ denote the means of the sample obtained from the three sets. Moreover, ${\bar{Y}}_{1}$, ${\bar{Y}}_{2}$ and ${\bar{Y}}_{3}$ denote the mean based on all units belonging to Set-I, II and III, respectively, and *k* denotes the total samples that can be drawn under the new sampling scheme.

Proof: By definition

(7) $${\bar{y}}_{msy}=\frac{k^{2}}{N}{\bar{y}}_{1}+\frac{\left(n-k-2\right)k}{N}{\bar{y}}_{2}+\frac{2k}{N}{\bar{y}}_{3}=\frac{1}{N}\left(k\sum_{i\in s_{1}}y_{1i}+k\sum_{i\in s_{2}}y_{2i}+k\sum_{i\in s_{3}}y_{3i}\right)\text{,}$$

where $s_{1}$, $s_{2}$ and $s_{3}$ stand for the sample obtained from the units of Set-I, II and III, respectively.

(8) $${\bar{y}}_{msy}=\frac{1}{N}\left(\sum_{i\in s_{1}}\frac{y_{1i}}{1/k}+\sum_{i\in s_{2}}\frac{y_{2i}}{1/k}+\sum_{i\in s_{3}}\frac{y_{3i}}{1/k}\right)=\frac{1}{N}\sum_{i\in s}\frac{y_{i}}{\pi_{i}}={\bar{y}}_{HT}\text{.}$$

The symbol ‘*s*’ in equation (8) denotes the sample drawn from the total units of population. Applying expectation on both sides of equation (7) gives:

(9) $$E\left({\bar{y}}_{msy}\right)=\frac{k^{2}}{N}E\left({\bar{y}}_{1}\right)+\frac{\left(n-k-2\right)k}{N}E\left({\bar{y}}_{2}\right)+\frac{2k}{N}E\left({\bar{y}}_{3}\right)\text{.}$$

Now,

$$E\left({\bar{y}}_{1}\right)=E\left(\frac{1}{k}\sum_{i=1}^{k}y_{1i}\right)=\frac{1}{k}\sum_{i=1}^{k}E\left(y_{1i}\right)\text{.}$$

(10) $$=E\left(\frac{1}{k}\sum_{i=1}^{k}y_{1i}\right)=E\left(y_{1i}\right)={\bar{Y}}_{1}$$

Similarly,

(11) $$E\left({\bar{y}}_{2}\right)=E\left(\frac{1}{n-k-2}\sum_{i=1}^{n-k-2}y_{2i}\right)=E\left(y_{2i}\right)={\bar{Y}}_{2}\text{,}$$

and,

(12) $$E\left({\bar{y}}_{3}\right)=E\left(\frac{1}{2}\left(y_{3i}+y_{3i}\right)\right)={\bar{Y}}_{3}\text{.}$$

Now using equations (10), (11), (12) in equation (9) and on simplification, we get $E\left({\bar{y}}_{msy}\right)=\bar{Y}$.

Now applying variance on both sides of equation (3) gives:

(13) $$Var\left({\bar{y}}_{msy}\right)=\frac{k^{4}}{N^{2}}Var\left({\bar{y}}_{1}\right)+\frac{{\left(n-k-2\right)}^{2}k^{2}}{N^{2}}Var\left({\bar{y}}_{2}\right)+\frac{4k^{2}}{N^{2}}Var\left({\bar{y}}_{3}\right)\text{,}$$

where,

(14) $$Var\left({\bar{y}}_{1}\right)=\frac{1}{k}\sum_{i=1}^{k}{\left({\bar{y}}_{1i}-{\bar{Y}}_{1}\right)}^{2}\text{,}$$

since each possible sample in Set-I has probability equal to *1/k*. Similarly,

(15) $$Var\left({\bar{y}}_{2}\right)=\frac{1}{k}\sum_{i=1}^{k}{\left({\bar{y}}_{2i}-{\bar{Y}}_{2}\right)}^{2}\text{,}$$

(16) $$Var\left({\bar{y}}_{3}\right)=\frac{1}{k}\sum_{i=1}^{k}{\left({\bar{y}}_{3i}-{\bar{Y}}_{3}\right)}^{2}\text{.}$$

Using equations (14), (15), (16) in equation (13), the variance of the mean ${\bar{y}}_{msy}$ under the suggested sampling scheme is obtained as:

(17) $$Var\left({\bar{y}}_{msy}\right)=\frac{1}{N^{2}}[k^{4}\left\{\frac{1}{k}\sum_{i=1}^{k}{\left({\bar{y}}_{1i}-{\bar{Y}}_{1}\right)}^{2}\right\}+{\left(n-k-2\right)}^{2}k^{2}\left\{\frac{1}{k}\sum_{i=1}^{k}{\left({\bar{y}}_{2i}-{\bar{Y}}_{2}\right)}^{2}\right\}+4k^{2}{\left({\bar{y}}_{3i}-{\bar{Y}}_{3}\right)}^{2}]\text{.}$$

Remark 1: Using the approach given by Sen-Yates-Grundy (Sen [24], Yates and Grundy [25]), the sampling variance equivalent to the variance in equation (17) may be obtained as:

(18) $$Var\left({\bar{y}}_{mdsy}\right)=\frac{1}{N^{2}}\left\{\frac{1}{2}\sum_{i=1}^{N}\sum_{\begin{array}{l} j=1\\ j\neq i \end{array}}^{N}\left(\pi_{i}\pi_{j}-\pi_{ij}\right){\left(\frac{y_{i}}{\pi_{i}}-\frac{y_{j}}{\pi_{j}}\right)}^{2}\right\}=Var_{SYG}\left({\bar{y}}_{HT}\right)$$

Remark 2: The Sen-Yates-Grundy type estimator of the sampling variance given in equation (18), is given as:

(19) $$var\left({\bar{y}}_{mdsy}\right)=\frac{1}{N^{2}}\left\{\frac{1}{2}\sum_{i=1}^{n}\sum_{\begin{array}{l} j=1\\ j\neq i \end{array}}^{n}\left(\frac{\pi_{i}\pi_{j}-\pi_{ij}}{\pi_{ij}}\right){\left(\frac{y_{i}}{\pi_{i}}-\frac{y_{j}}{\pi_{j}}\right)}^{2}\right\}=var_{SYG}\left({\bar{y}}_{HT}\right)$$

One can use the mathematical expressions of $\pi_{i}$ and $\pi_{ij}$ given in equation (1) and equation (2) in equations (18), (19) to get the sampling variance as well as its unbiased estimator based on the proposed method.

## 3 Linear trend

Linear trend refers to the arithmetic progression which may be found in the order in which the population units are arranged, which may be in increasing or decreasing pattern. One can observe a moderate to a high degree of linear trend in many practical cases. As an illustration, educational institutes almost everywhere in the world offer admissions on merit basis in various academic departments. Many universities tend to allocate roll numbers to their students on the basis on their quantified academic scores during admission process. In such cases, intelligent students tend to occupy the top enrollment numbers. Thus, upon admission, if the examination marks obtained by students are observed in order of students’ enrollment roll numbers, one can expect a moderate level of increasing linear trend since the top-enrolled students, being the merit toppers, tend to perform better in subsequent examinations.

Likewise, to practically observe a decreasing form of the linear trend, one can think of the daily record of the milk yield data, starting from calving. One can naturally expect that the daily milk yield quantity will tend to decrease with the passage of time, thus resulting in a decreasing type of linear trend.

Suppose *N* = *nk* = *k∙k* + *(n-k-1)k* + *k* population units exhibit a linear trend. Thus,

(20) $$y_{i}=a+bi,where\;i=1,2,3,\ldots .,N\text{.}$$

The sampling variance under simple random sampling based on perfect linear trend defined in equation (20) is:

(21) $$Var\left({\bar{y}}_{r}\right)=\left(k-1\right)\left(N+1\right)\frac{b^{2}}{12}$$

The sampling variance under systematic random sampling design is as follows:

(22) $$Var\left({\bar{y}}_{sy}\right)=\left(k-1\right)\left(k+1\right)\frac{b^{2}}{12}$$

Further, the variance in the case of diagonal systematic sampling method is as follows:

(23) $$Var\left({\bar{y}}_{dsy}\right)=\left(k-n\right)\left[n\left(k-n\right)+2\right]\frac{b^{2}}{12n}$$

where *N* = *nk* + *r*. The variance under the modified systematic sampling suggested by Subramani [7] is given as:

(24) $$Var\left({\bar{y}}_{ssy}\right)=\left(\frac{{\left(n-1\right)}^{2}+1}{n^{2}}\right)\left(k-1\right)\left(k+1\right)\frac{b^{2}}{12}$$

The variance of the Azeem et al. [22] sampling scheme is given as:

(25) $$Var\left({\bar{y}}_{mdsy}\right)={\left(\frac{n-k}{n}\right)}^{2}\left(k-1\right)\left(k+1\right)\frac{b^{2}}{12}$$

Finally, under complete linear trend in the population units, the variance of the suggested sampling scheme can be derived as:

(26) $$Var\left({\bar{y}}_{msy}\right)=w_{1}^{2}Var\left({\bar{y}}_{1}\right)+w_{2}^{2}Var\left({\bar{y}}_{2}\right)+w_{3}^{2}Var\left({\bar{y}}_{3}\right)$$

In order to obtain the variance of the proposed sampling scheme under perfect linear trend, we first need to obtain the variance expressions for ${\bar{y}}_{1}$, ${\bar{y}}_{2}$ and ${\bar{y}}_{3}$. Since the total number of units in Set-I are *k* × *k* = *k*^*2*^, so using *k* = *n* in equation (23) leads to

(27) $$Var\left({\bar{y}}_{1}\right)=0$$

Moreover, since a linear systematic sampling design is used in both Set-II and Set-III and since the right-hand side of equation (22) does not depend on *n*, so,

(28) $$Var\left({\bar{y}}_{2}\right)=\left(k-1\right)\left(k+1\right)\frac{b^{2}}{12}$$

Similarly,

(29) $$Var\left({\bar{y}}_{3}\right)=\left(k-1\right)\left(k+1\right)\frac{b^{2}}{12}$$

Substituting equations (27), (28), (29) in equation (26), and after simplification, the variance of ${\bar{y}}_{msy}$ is obtained as:

(30) $$Var\left({\bar{y}}_{msy}\right)=\left[\frac{{\left(n-k-2\right)}^{2}+4}{n^{2}}\right]\left(k-1\right)\left(k+1\right)\frac{b^{2}}{12}\text{.}$$

## 4 Efficiency comparison under perfect linear trend

### 4.1 Comparison with simple random and systematic random sampling

In circumstances where the units exhibit a perfect linear tendency, the suggested sampling scheme will be more efficient compared to the simple random sampling design if,

(31) $$Var\left({\bar{y}}_{msy}\right)<Var\left({\bar{y}}_{r}\right)$$

Substituting equations (21) and (30) in equation (31) yields:

(32) $$\left[\frac{{\left(n-k-2\right)}^{2}+4}{n^{2}}\right]\left(k+1\right)<N+1\text{.}$$

Condition (32) is strong and always holds since N=nk>k. This means that the suggested sampling procedure is more efficient than the simple random sampling procedure.

Our suggested sampling technique will be more efficient than the systematic random sampling design if

(33) $$Var\left({\bar{y}}_{msy}\right)<Var\left({\bar{y}}_{sy}\right)$$

Substituting equations (22), (30) in equation (33) yields:

$$\left[\frac{{\left(n-k-2\right)}^{2}+4}{n^{2}}\right]\left(k-1\right)\left(k+1\right)\frac{b^{2}}{12}<\left(k-1\right)\left(k+1\right)\frac{b^{2}}{12}\text{.}$$

The above inequality on further simplification reduces to:

(34) $$\left[\frac{{\left(n-k-2\right)}^{2}+4}{n^{2}}\right]<1\text{,}$$

Condition (34) is strong, thus the suggested method is more efficient compared to systematic random sampling.

### 4.2 Comparison with Subramani's [7] modified systematic sampling

Under perfect linear trend, the new suggested sampling scheme will be more efficient than the sampling design suggested by Subramani [7] if,

(35) $$Var\left({\bar{y}}_{msy}\right)<Var\left({\bar{y}}_{ssy}\right)$$

Using equations (24), (30) in equation (35) yields:

$$\left[\frac{{\left(n-k-2\right)}^{2}+4}{n^{2}}\right]\left(k-1\right)\left(k+1\right)\frac{b^{2}}{12}\;<\;\left(\frac{{\left(n-1\right)}^{2}+1}{n^{2}}\right)\left(k-1\right)\left(k+1\right)\frac{b^{2}}{12}\text{,}$$

Or

$$\left[\frac{{\left(n-k-2\right)}^{2}+4}{n^{2}}\right]\;<\;\left(\frac{{\left(n-1\right)}^{2}+1}{n^{2}}\right)\text{,}$$

Or

(36) $${\left(n-k-2\right)}^{2}+3<{\left(n-1\right)}^{2}\text{.}$$

Condition (36) always holds. This implies that the suggested method is always more efficient than Subramani's [7] modified sampling.

### 4.3 Comparison with diagonal systematic sampling

Our new proposed sampling design will be more precise than the diagonal sampling design if,

(37) $$Var\left({\bar{y}}_{msy}\right)<Var\left({\bar{y}}_{dsy}\right)$$

Using equations (23), (30) in equation (37)

$$\left[\frac{{\left(n-k-2\right)}^{2}+4}{n^{2}}\right]\left(k-1\right)\left(k+1\right)\frac{b^{2}}{12}\;<\;\left(k-n\right)\left[n\left(k-n\right)+2\right]\frac{b^{2}}{12n}$$

Or

$$\left[\frac{{\left(n-k-2\right)}^{2}+4}{n}\right]\left(k-1\right)\left(k+1\right)<\;\left(k-n\right)\left[n\left(k-n\right)+2\right]\text{,}$$

Or

(38) $$\left[{\left(n-k-2\right)}^{2}+4\right]\left(k-1\right)\left(k+1\right)<\;n\;\left(k-n\right)\left[n\left(k-n\right)+2\right]$$

Since the proposed method uses *n* > *k*, which implies condition (38) is strong and always holds.

### 4.4 Comparison with Azeem et al. [22] Sampling design

Our proposed sampling scheme will be more efficient than Azeem et al. [22] sampling design if,

(39) $$Var\left({\bar{y}}_{msy}\right)<Var\left({\bar{y}}_{mdsy}\right)$$

Using equations (25), (30) in equation (39) gives:

$$\left[\frac{{\left(n-k-2\right)}^{2}+4}{n^{2}}\right]\left(k-1\right)\left(k+1\right)\frac{b^{2}}{12}\;<\;{\left(\frac{n-k}{n}\right)}^{2}\left(k-1\right)\left(k+1\right)\frac{b^{2}}{12}\text{,}$$

Or

$${\left(n-k-2\right)}^{2}+4<\;{\left(n-k\right)}^{2}\text{,}$$

Or

(40) $${\left(m-2\right)}^{2}+4<\;m^{2},where\;m=n-k$$

Since the proposed method uses n>k, so condition (40) is strong and always hold for m=n−k>2⇒n>k+2.

## 5 Efficiency comparison using milk-yield data

Efficiency comparison is a useful tool to know the usefulness of any statistical technique. Yang et al. [26] utilized efficiency comparison method for ranked set sampling for a modified geometric distribution. The improvement in efficiency of the proposed method over the existing sampling techniques is assessed using the milk yield data taken from Pandey and Kumar [27] and is presented in Table 6 (see Appendix). The results in Table 2 clearly indicate that our suggested sampling scheme is more precise than the popularly used sampling schemes. The milk-yield (measured in liters) related to the Sahiwal cows for a 252 consecutive days period, starting from the day of calving, has been considered from the paper of Pandey and Kumar [27]. One can clearly observe a decreasing linear tendency in the data where the milk yield follows a decreasing pattern over time. The variances of various systematic sampling methods for data set are presented in Table 2. The findings clearly indicate that our new suggested sampling procedure is more precise than the existing sampling designs discussed in Section-3. As the population size in the milk yield data is 252 and since systematic random sampling scheme needs N=kn, so for various choices of *n* and *k*, a few units from our population in the milk yield data were randomly removed in order to compromise between the choice of values of *n, N*, and *k*. As an example, if we choose *n* = 10 with *k* = 25, we delete two population units at random in order to reduce the population size to 250 units in place of using *N* = 252 units. This will make efficiency comparison feasible for milk yield data.

**Table 2:** Calculations of variances of various sampling designs using milk yield data set.

*n*	*k*	$Var\left({\bar{y}}_{r}\right)$	$Var\left({\bar{y}}_{sy}\right)$	$Var\left({\bar{y}}_{dsy}\right)$	$Var\left({\bar{y}}_{ssy}\right)$	$Var\left({\bar{y}}_{mdsy}\right)$	$Var\left({\bar{y}}_{msy}\right)$
83	3	1.7329	2.0410	1.6124	0.9154	0.8653	0.6723
63	4	1.5051	1.1436	1.0070	0.7900	0.5180	0.3874
49	5	1.0628	0.7286	0.6023	0.5826	0.3496	0.2317
42	6	0.9291	0.6542	0.5317	0.4604	0.2604	0.1831
35	7	0.7690	0.5881	0.4781	0.3713	0.2267	0.1426
31	8	0.7157	0.5017	0.4496	0.3418	0.1807	0.1241
28	9	0.6143	0.4210	0.3305	0.2501	0.1268	0.1004
25	10	0.5243	0.3781	0.2851	0.1947	0.1097	0.0872
22	11	0.5034	0.3535	0.2641	0.1693	0.0988	0.0686
21	12	0.4632	0.3130	0.2345	0.1471	0.0923	0.0713
19	13	0.4153	0.3042	0.2160	0.1260	0.0891	0.0638
18	14	0.3613	0.2896	0.1990	0.1063	0.0856	0.0609
16	15	0.3650	0.2775	0.1630	0.0993	0.0833	0.0581

Next let us consider the cases in which a perfect linear trend is followed by the units of a finite population. Efficiency comparison has been conducted for different choices of the values of *N*, *n,* and *k*. The variances of the sample mean of the suggested and a few other popular sampling designs have been given in Table 3. The different values of the sample and population sizes for the purpose of efficiency comparison were taken in a manner so that N=kn where n>k. It is also to be noted that the constant $b^{2}$ is a multiplication factor in the variance expressions of all of the sampling procedures discussed in Section 3, so in order to make the efficiency comparison analysis simple, *b* = 1 has been used in the calculation of the variances in Table 3. The findings from Table 3 clearly indicate that our proposed systematic sampling scheme is superior in terms of efficiency over the existing sampling schemes, including the one suggested by Azeem et al. [22].

**Table 3:** Linear trend – based variances of different sampling designs.

*n*	*k*	$Var\left({\bar{y}}_{r}\right)$	$Var\left({\bar{y}}_{sy}\right)$	$Var\left({\bar{y}}_{dsy}\right)$	$Var\left({\bar{y}}_{ssy}\right)$	$Var\left({\bar{y}}_{mdsy}\right)$	$Var\left({\bar{y}}_{msy}\right)$
10	4	10.25	1.25	2.90	1.03	0.45	0.25
	6	25.42	2.92	1.27	2.39	0.47	0.23
	8	47.25	5.25	0.30	4.31	0.21	0.21
30	5	50.33	2.00	51.94	1.87	1.39	1.18
	10	225.75	8.25	33.22	7.72	3.67	3.01
	15	526.17	18.67	18.67	17.46	4.67	3.59
	20	951.58	33.25	8.28	31.11	3.69	2.51
	25	1502.00	52.00	2.06	48.65	1.44	0.75
50	10	375.75	8.25	133.20	7.93	5.28	4.78
	20	1584.92	33.25	74.90	31.95	11.97	10.48
	30	3627.42	74.92	33.27	71.98	11.99	9.83
	40	6503.25	133.25	8.30	128.03	5.33	3.62
100	20	3168.25	33.25	533.20	32.59	21.28	20.24
	40	13003.25	133.25	299.90	130.61	47.97	44.88
	60	29504.92	299.92	133.27	293.98	47.99	43.43
	80	52673.25	533.25	33.30	522.69	21.33	17.49
500	100	412508.25	833.25	13333.20	829.92	533.28	527.97
	200	1658349.92	3333.25	7499.90	3319.94	1199.97	1184.08
	300	3737524.92	7499.92	3333.27	7469.98	1199.99	1176.23
	400	6650033.25	13333.25	833.30	13280.02	533.33	512.42

## 6 Simulation study

**Fig. 1:**
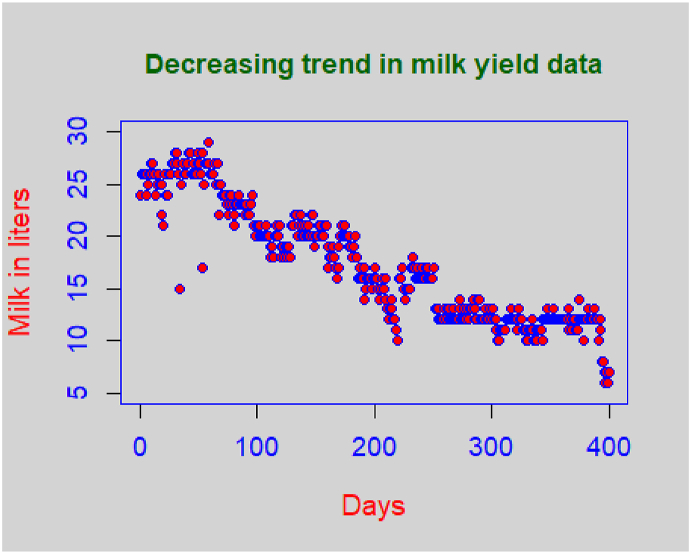
Linear trend in milk yield data.

**Fig. 2:**
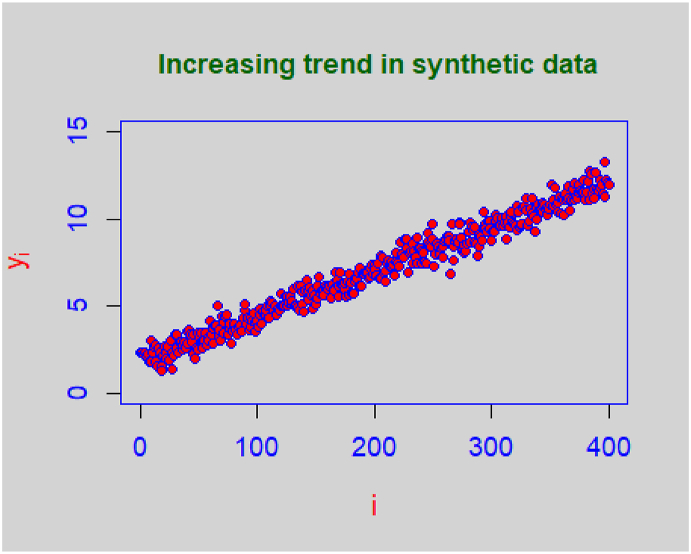
Linear trend in artificial data.

A simulation study was carried out to compare the performance of the proposed sampling design with the Azeem et al. [22] sampling design. We used two data sets for simulation – a real data set and an artificial data set. The real data set was taken from Pandey and Kumar [27] which exhibits a decreasing trend, as shown in Fig. 1. Besides the real population, an artificial population of 400 units with a non-exact linear trend was generated, as shown in Fig. 2.

The results of simulated variances of the mean under the proposed and the Azeem et al. [22] sampling designs for the real and artificial data sets have been presented in Table 4 and Table 5, respectively. The real data set on milk yield for 420 days was taken from Pandey and Kumar [27] and is presented in Table 7 (see Appendix). The results presented in the tables were averaged over 1000 iterations. It is also to be noted that in order to reconcile between the population and sample sizes, a few values were randomly deleted to make calculations possible. One may clearly observe the improvement in efficiency over the Azeem et al. [22] systematic sampling design.

**Table 4:** Simulated variances based on milk yield data.

*k*	*n*	$Var\left({\hat{\mu }}_{msy}\right)$	$Var\left({\hat{\mu }}_{mdsy}\right)$
10	40	0.07020625	0.07897375
	39	0.06506246	0.0687879
	38	0.0676392	0.07166136
	37	0.06648035	0.06871658
9	43	0.0623855	0.06300824
	42	0.0646646	0.06662195
	41	0.06323439	0.06426209
	35	0.08303383	0.08589805
8	50	0.01932045	0.02289855
	47	0.02149887	0.02213083
	46	0.0197552	0.02069282
	25	0.072272	0.0769128
7	55	0.01034132	0.01279601
	53	0.01082763	0.01154477
	45	0.0120892	0.01298613
	42	0.01400712	0.01565078
6	65	0.03050977	0.03143663
	62	0.02805151	0.03172945
	60	0.02847568	0.02886475
	55	0.03090053	0.03379904
5	80	0.01470031	0.01726031
	77	0.01508396	0.01544109
	65	0.02651124	0.02723342
	62	0.0301295	0.0331334

**Table 5:** Simulated variances based on synthetic data.

*k*	*n*	$Var\left({\hat{\mu }}_{msy}\right)$	$Var\left({\hat{\mu }}_{mdsy}\right)$
10	40	0.00349539	0.004362683
	39	0.003673637	0.003827209
	37	0.003856536	0.0038967
	35	0.003797756	0.004148683
9	43	0.007839106	0.008811967
	42	0.008341945	0.008632896
	41	0.008479745	0.008992375
	35	0.009368519	0.01024975
8	48	0.007535187	0.008435035
	45	0.00725946	0.008845018
	43	0.006945541	0.00778288
	38	0.008121302	0.008583599
7	55	0.003963771	0.005508161
	53	0.003644271	0.003992082
	50	0.00321205	0.003999207
	40	0.004209553	0.004506942
6	65	0.01133966	0.01206911
	62	0.0102278	0.0116993
	60	0.01012279	0.01077084
	55	0.006761836	0.009083443
5	80	0.003063108	0.003224376
	77	0.002667662	0.002964467
	70	0.00191757	0.002318078
	63	0.001259042	0.001458852

## 7 Conclusion

Unlike the Azeem et al. [22] sampling design which divides the entire population into two disjoint and exhaustive subgroups, the suggested sampling scheme partitions the finite population into three mutually exclusive subsets. For the purpose of sample selection, the new proposed sampling design utilizes the diagonal systematic sampling method in Set-I, and a linear systematic sampling method in the other two sets. A weighting approach is then used to estimate the finite population mean based on the new sampling scheme. Efficiency comparison analysis has been carried out to study the performance of the new sampling scheme with other existing sampling schemes, using a real data set as well as perfect linear trend, and the improvement in efficiency has been shown. Based on the empirical and theoretical analysis in the current study, the proposed method is recommended to be utilized in those practical circumstances where a high degree of an increasing or decreasing linear trend exists in the population.

Our analysis showed the improvement in efficiency over the existing sampling schemes for the mean estimator. For future research, it may be interesting if researchers study the efficiency in estimators of population variance under the proposed and the available sampling schemes.

## Author contribution statement

Muhammad Azeem, Ph.D.: Conceived and designed the experiments; Performed the experiments; Wrote the paper.

Sundus Hussain: Performed the experiments; Analyzed and interpreted the data.

Musarrat Ijaz: Performed the experiments; Contributed reagents, materials, analysis tools or data.

Najma Salahuddin: Analyzed and interpreted the data; Contributed reagents, materials, analysis tools or data.

Abdul Salam: Conceived and designed the experiments; Contributed reagents, materials, analysis tools or data.

## Data availability statement

Data included in article/supp. material/referenced in article.

## Funding statement

The authors received no funding for this study.

## Declaration of competing interest

The authors declare that they have no known competing financial interests or personal relationships that could have appeared to influence the work reported in this paper.

## Figures and Tables

**Table 6:** Horizontally Day wise Milk-Yield Data Set for S-19 brand of Cows (Pandey and Kumar [27])

10	10	10	10	10	10	10	10	10	10	10	10	11	12	12	12
12	12	12	12	12	12	12	13	12	12	12	12	12	12	13	11
12	12	12	12	14	13	13	13	13	13	13	12	12	12	12	13
13	12	12	13	12	12	12	12	13	12	12	12	12	12	12	12
12	12	12	12	13	12	12	12	12	12	12	12	12	13	12	12
12	12	13	12	13	12	12	12	12	12	12	12	12	12	12	12
12	11	10	14	15	14	14	13	14	14	12	14	14	14	13	14
12	14	13	14	14	14	13	14	14	14	13	14	13	12	13	13
12	12	12	12	12	12	13	13	15	12	13	12	12	12	12	12
11	11	12	12	12	12	12	12	12	12	13	12	12	12	12	12
12	12	12	13	13	13	14	15	14	14	14	12	14	14	14	12
13	12	12	12	12	13	12	12	13	12	12	12	11	10	11	11
11	11	11	10	10	10	11	11	10	10	10	12	12	12	12	12
12	12	12	13	13	13	14	15	14	14	14	12	14	14	14	12
12	12	12	12	10	11	9	8	8	8	8	8	8	9	8	8
8	8	8	8	8	9	8	8	8	9	8	9	8	8	8	8
8	8	8	8	8	8	8	9	9	9	9	9	9	8	8	8
8	9	8	3	2	2	1	0								

**Table 7:** Horizontally Day wise Milk Yield Data (in Liters) for X-205 brand of Cows for 420 days

8	10	8	8	17	18	18	18	18	18	18	18	18	20	24	24
24	26	26	26	26	24	25	26	27	26	27	26	24	24	25	26
25	25	22	21	26	24	24	24	26	26	27	27	27	28	28	27
26	15	25	27	27	26	26	27	27	28	28	26	26	27	26	26
27	28	27	26	28	17	25	27	27	27	29	26	26	26	26	27
25	27	22	25	25	24	24	24	24	23	22	23	24	24	23	22
21	23	23	24	23	23	23	23	22	23	23	22	22	23	23	24
21	20	20	21	21	21	20	20	20	20	21	20	20	20	18	19
19	18	20	20	21	20	21	19	18	19	18	19	19	18	19	18
21	21	21	22	22	20	20	21	22	21	20	20	21	20	20	21
20	22	22	20	19	20	20	20	21	21	20	20	20	21	21	17
19	18	18	19	18	18	17	16	17	19	20	21	20	21	20	20
20	20	19	19	18	19	20	20	18	16	16	17	16	16	17	14
15	16	16	16	16	15	16	16	17	16	15	14	15	15	16	15
16	14	13	12	13	14	13	12	12	11	11	10	16	16	17	17
15	14	15	15	15	15	17	17	18	17	16	16	17	16	16	16
17	16	16	17	17	16	16	16	16	16	17	13	13	13	12	12
12	12	12	13	12	13	12	13	12	12	13	12	12	12	12	14
13	12	12	13	12	12	13	12	13	13	13	14	13	12	12	14
14	14	12	12	12	13	13	12	12	13	13	12	13	12	11	12
10	10	11	11	11	11	11	12	12	12	12	13	12	12	12	12
12	11	13	12	12	12	11	11	11	10	10	11	11	12	11	10
10	11	10	11	11	11	10	12	12	12	12	13	12	12	12	13
12	12	12	12	12	12	12	12	12	12	12	12	13	11	12	11
12	12	12	11	11	14	12	12	12	10	12	12	12	13	12	12
12	12	13	13	12	12	10	11	12	8	8	7	6	7	6	7
6	5	4	1												
